# Cohort study of prediction of venous thromboembolism in emergency department patients with extremity symptoms

**DOI:** 10.1007/s11739-024-03696-3

**Published:** 2024-07-02

**Authors:** Anders Gottsäter, Ulf Ekelund, Olle Melander, Anders Björkelund, Bodil Ohlsson

**Affiliations:** 1 Department of Clinical Sciences in Malmö, University of Lund, S-20502 Malmö, Sweden; 2 Department of Emergency and Internal Medicine, Skåne University Hospital, S-20502 Malmö, Sweden; 3 Department of Clinical Sciences in Lund, University of Lund, S-22100 Lund, Sweden; 4 Department of Emergency and Internal Medicine, Skåne University Hospital, S-22242 Lund, Sweden; 5 Centre for Environmental and Climate Research, University of Lund, S-22100 Lund, Sweden

**Keywords:** Venous thromboembolism, Deep vein thrombosis, Pulmonary embolism, Prediction

## Abstract

Despite diagnostic algorithms, identification of venous thromboembolism (VTE) in emergency departments (ED) remains a challenge. We evaluated symptoms, background, and laboratory data in 27,647 ED patients presenting with pain, swelling, or other symptoms from the extremities, and identified predictors of VTE diagnosis within one year. Predictors of a clinical decision to perform phlebography, ultrasound, or computer tomography (CT) angiography of pelvic, lower, or upper extremity veins, CT of pulmonary arteries, or pulmonary scintigraphy at the ED or within 30 days, and the results of such investigations were also evaluated. A total of 3195 patients (11.6%) were diagnosed with VTE within one year. In adjusted analysis of patients in whom all laboratory data were available, a d-dimer value ≥ 0.5 mg/l (odds ratio [OR]: 2.602; 95% confidence interval [CI] 1.894–3.575; *p* < 0.001) at the ED and a previous diagnosis of VTE (OR: 6.037; CI 4.465–8.162; *p* < 0.001) independently predicted VTE within one year. Of diagnosed patients, 2355 (73.7%) had undergone imaging within 30 days after the ED visit and 1730 (54.1%) were diagnosed at this examination. Lower age (OR: 0.984; CI 0.972–0.997; *p* = 0.014), higher blood hemoglobin (OR: 1.023; CI 1.010–1.037; *p* < 0.001), C-reactive protein (OR: 2.229; CI 1.433–3.468; *p* < 0.001), d-dimer (OR: 8.729; CI 5.614–13.574; *p* < 0.001), and previous VTE (OR: 7.796; CI 5.193–11.705; *p* < 0.001) predicted VTE on imaging within 30 days, whereas female sex (OR 0.602 [95% CI 0.392–0.924]; *p* = 0.020) and a previous diagnosis of ischemic heart disease (OR 0.254 [95% CI 0.113–0.571]; *p* = 0.001) were negative predictors of VTE. In conclusion, analysis of 27,647 ED patients with extremity symptoms confirmed the importance of well-established risk factors for VTE. Many patients developing VTE within one year had initial negative imaging, highlighting the importance of continued symptom vigilance.

## Introduction

The high patient inflow to emergency departments (EDs) necessitates rapid and accurate selection of ED patients for acute treatment and in-hospital care [1, 2]. Identification of variables collected at the ED predicting significant morbidity and mortality is a clinical challenge. Digital technologies have been developed to improve clinical decision making [3, 4], but the potential applications of such methods in the ED remain unclear [5].

Many patients present to the ED with different extremity symptoms, signs, and clinical risk factors suggesting deep venous thrombosis (DVT), but these variables cannot be individually used to confirm the diagnosis. Despite the availability of several different diagnostic algorithms [6–8] such as different versions of the Wells score [9–11], the recent European DVT guidelines [12] pointed out remaining diagnostic problems in patients with suspected extremity DVT.

The primary endpoint of the present study was to evaluate predictors of a diagnosis of venous thromboembolism (VTE), either extremity DVT or pulmonary embolism (PE) within one year in ED patients with extremity symptoms. Secondary outcomes were to evaluate predictors of diagnostic imaging within 30 days, and imaging results. Such knowledge may potentially help later development of artificial intelligence (AI) and machine learning-based diagnostic algorithms [13].

## Methods

### Subjects

All patients aged ≥ 18 years visiting an ED in Region Skåne´s hospitals in Ängelholm, Helsingborg, Hässleholm, Kristianstad, Landskrona, Lund and Malmö (Skåne University Hospital), Trelleborg, and Ystad during 2017–2018 for pain, swelling, or unspecified symptoms from the extremities such as heaviness, pressure or discomfort, as classified by the triage nurse according to the Rapid Emergency Triage and Treatment System (RETTS) system [14, 15], were included. Patients with rashes or symptoms after extremity trauma were classified in other categories and excluded from this study. In case of more than one visit during the period, the first visit was used in the analysis. Patients residing outside Sweden on December 31, 2016, were excluded during 2017, and those residing outside Sweden on December 31, 2017, were excluded during 2018 as they could not be followed up in national registries. The Wells score [9–11] is recommended for use at all study hospitals when lower extremity DVT is suspected. Negative imaging is not routinely repeated, but patients are prompted to return for recurrent symptoms.

### Baseline data

Data on age, sex, and symptom category according to RETTS [14, 15] were collected at the ED visit, together with results of relevant blood test results within 24 h; blood (b-) hemoglobin, plasma (p-) C-reactive protein (CRP), b-platelet and leukocyte counts, p-d-dimer, and p-creatinine.

We also collected data on background variables with potential relationship to VTE from the Inpatient and Outpatient Registers at the National Board of Health and Welfare (http://socialstyrelsen.se/english) [16] using the International Classification of Diseases (ICD) revision 10. The following variables were included in the analysis: in- or outpatient surgery within 30 days before the visit, comorbidities registered within five years before the ED visit, such as malignancy (C00-C96), hypertensive disease (I10-16), ischemic heart disease (I20-I25), other heart disease (I30-I50), cerebrovascular diseases (I60-I69), VTE (I26, I80-I82), coagulation diseases (D65-69), inflammatory diseases (M04-M14, M30-M36), alcohol abuse (F10), and diabetes mellitus (E08-E13).

Data on ongoing medication at the ED visit with potential relationship to VTE were collected from the Prescribed Drug Register at the National Board of Health and Welfare [17]; anticoagulation and antiplatelet (ATC codes B01), lipid lowering (ATC codes C10), glucose lowering (ATC codes A10), blood pressure lowering (ATC codes C02, C03, C08, and C09), immunosuppressive (ATC codes H02, L01, L04), and estrogen (ATC codes G03A, G03C, and L02A) treatment.

Data on the following diagnostic imaging performed at and during 30 days after the ED visit were collected from hospital files: phlebography, ultrasound, or computer tomography (CT) angiography of veins in the pelvic region, lower and upper extremities, CT of pulmonary arteries, and pulmonary scintigraphy.

### Outcome variables

The primary outcome variable was a diagnosis of VTE, i.e. extremity DVT or PE, within one year of the ED visit registered in the Inpatient Register, Outpatient Register, or Cause of Death Register at the National Board of Health and Welfare (http://socialstyrelsen.se/english) [16]. The Inpatient Register uses ICD revision 10 codes for classification of diagnoses, and diagnoses of DVT in the extremity circulation (I80.1, I80.2, I80.3, I80.8, I80.9) or PE (I26) were considered relevant for this study.

Secondary outcome variables were a clinical decision to perform diagnostic imaging at or within 30 days after the ED visit, and the results of the performed investigations.

### Statistics

Age and most blood test results were normally distributed. CRP and d-dimer were not normally distributed and therefore categorized as pathological at CRP ≥ 5 mg/l or d-dimer ≥ 0.5 mg/l [18]. Values < 0.1 mg/l in d-dimer were set to 0.1 mg/l and CRP values < 0.6 mg/l were set to 0.6 mg/l. Logistic regression was employed to evaluate which variables were independent predictors of imaging for suspected VTE within 30 days and results of such imaging as dependent variables. As they were highly correlated with previous and concomitant diseases, data on medication were not entered in these calculations.

For comorbidities, logistic regression was first performed with each disease, adjusted for sex and age. Diseases not associated with the dependent variable were excluded from further calculations, i.e., previous stroke was excluded from the calculations regarding imaging, and previous stroke, alcoholism, and coagulation disorders were excluded from calculations regarding VTE diagnosis at imaging. After crude calculation of the independent variables sex, age, symptoms, laboratory parameters, recent surgery, and concomitant diseases, adjusted regressions were performed for all parameters statistically associated with the dependent variables in the crude calculations and expressed as odds ratio (OR) and 95% confidence interval (CI). Variables not statistically associated with the dependent variables were excluded and are not shown in the tables. As all patients did not undergo all laboratory investigations, adjusted OR were only calculated in patients in whom all laboratory data were available.

Cox regression was performed to calculate the risk to be diagnosed with VTE within one year after the baseline ED visit. After calculations of crude hazard ratio (HR) and 95% CI, adjusted hazard ratio (HR) was calculated with parameters statistically associated with the dependent variables in the crude calculations.

In patients negative at imaging within 30 days, Mann–Whitney *U* test or Independent Samples t test for continuous variables, and Fisher’s exact test for categorical variables were used to calculate differences between those with or without VTE between 30 days and one year.

Values are expressed as number and percentage, mean ± standard deviation (SD) or median and interquartile ranges (IQR). *P*-values < 0.05 were considered significant. Statistical calculations were performed in SPSS, version 28.

### Ethical approval

The study was approved by the Swedish Ethical Review Authority (2019–05783). The requirement for informed consent was waived by the Swedish Ethical Review Authority because of the retrospective nature of the study. All methods were carried out in accordance with relevant guidelines and regulations. All analyses were made on anonymized datasets and no individual person’s data are presented in the manuscript.

## Results

### Prediction of VTE within one year after the ED visit

**Fig. 1:**
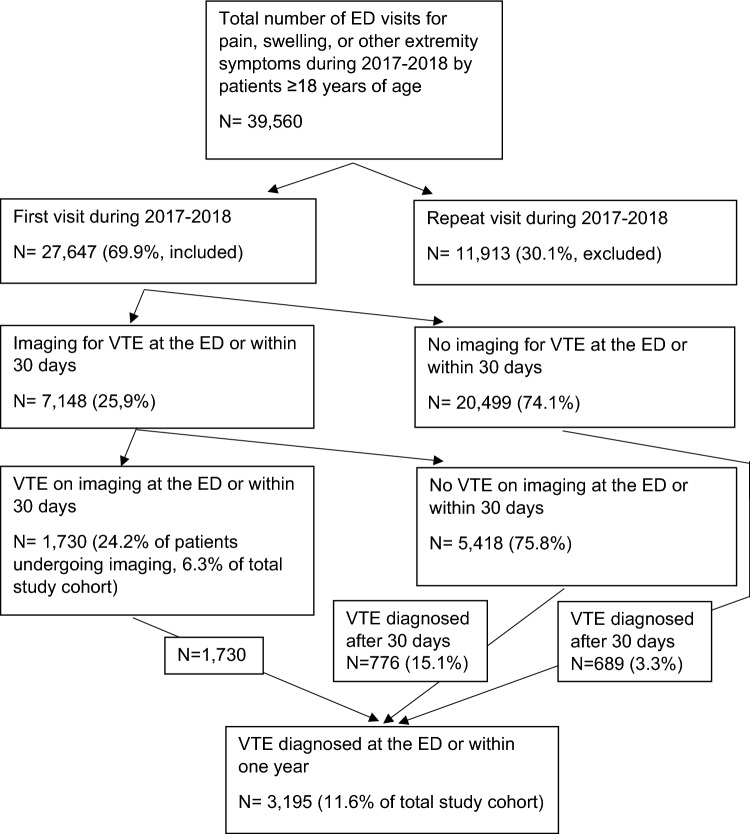
Study flow chart. ED, emergency department; VTE, venous thromboembolism

From January 1, 2017 to December 31, 2018, 39,560 visits by 27,647 individual patients aged ≥ 18 years caused by extremity symptoms were registered at the Region Skåne EDs (Fig. 1). Of these, 15,273 (55%), 4265 (15%), and 8,109 (29%) were classified in the categories pain, swelling, and unspecified symptoms, respectively. Among all 27,647 patients, 3,195 (11.6%) were diagnosed with VTE within one year of the initial ED visit. Table 1 shows baseline data and the imaging results at the ED or within 30 days in relation to a final diagnosis of VTE within one year. Table 2 shows that a d-dimer value ≥ 0.5 mg/l (HR 2.602 [95% CI 1.894–3.575]; *p* < 0.001) and a previous diagnosis of VTE (HR 6.037 [95% CI 4.465–8.162]; *p* < 0.001) were the only independent predictors of a diagnosis of VTE within one year after the initial ED visit. Out of the 3195 patients diagnosed with VTE within one year, 2355 (73.7%) had undergone imaging within 30 days after the ED visit and 1730 (54.1%) had received a VTE diagnosis at this examination (Fig. 1). Among patients with negative imaging results during the first 30 days after the ED visit 776 (15.1%) were diagnosed with VTE later within one year, and among those not undergoing imaging during the first 30 days, this proportion was 689 (3.3%).

**Table 1:** Baseline data and results of radiological imaging at and during 30 days after the emergency department visit in patients with and without a final diagnosis of venous thromboembolism during one-year follow-up among 27,647 patients presenting at an emergency department with extremity symptoms

Baseline variable	VTE diagnosis within one year (*n =* 3195)	No VTE diagnosis within one year (*n =* 24,452)
Age (years)	64.1 ± 16.9	59.5 ± 19.6
Male sex	1618 (50.6)	11,649 (47.6)
Symptom category pain	1309 (41.0)	13,964 (57.1)
Symptom category swelling	776 (24.3)	3489 (14.3)
Symptom category unspecified	1110 (34.7)	6999 (28.6)
B-hemoglobin (g/l)	136 ± 18 (*n =* 2692)	134 ± 18 (*n =* 10,674)
P–C-reactive protein (mg/l)	7.4 (2.0–31.0, *n =* 1363	6.9 (2.1–28.0, *n =* 11,632)
B-leukocyte count (× 10^9^/l)	8.9 ± 9.0 (*n =* 1882)	9.3 ± 4.8 (*n =* 8369)
B-platelet count (× 10^9^/l)	250 ± 88 (*n =* 2364)	257 ± 88 (*n =* 7015)
P-creatinine (micromol/l)	83 ± 38 (*n =* 2694)	88 ± 61 (*n =* 10,710)
P-D-dimer (mg/l)	0.14 (0.10–0.41, *n =* 270)	0.15 (0.10–0.38, *n =* 2541)
Recent (30 days) outpatient surgery	461 (14.4)	3264 (13.3)
Recent (30 days) inpatient surgery	314 (9.8)	1379 (5.6)
Comorbidities (ICD codes)		
Malignancy (C)	631 (19.7)	3042 (12.4)
Hypertensive disease (I10-16)	783 (24.5)	6005 (24.6)
Ischemic heart disease (I20-25)	255 (8.0)	2406 (9.8)
Other heart disease (I30-50)	401 (12.6)	4202 (17.2)
Cerebrovascular disease (I60-69)	149 (4.7)	1332 (5.4)
VTE (I26, 80–82)	2301 (72.0)	1409 (5.8)
Coagulation diseases (D65-69)	103 (3.2)	428 (1.8)
Inflammatory disease (M04-14, 30–36)	249 (7.8)	2464 (10.1)
Alcohol abuse (F10)	68 (2.1)	693 (2.8)
Diabetes mellitus (E08-13)	268 (8.4)	2563 (10.5)
Ongoing medication (ATC codes)		
Anticoagulation or antiplatelet (B01)	1377 (43.1)	6090 (24.9)
Lipid lowering (C10)	466 (14.6)	4357 (17.8)
Glucose lowering (A10)	241 (7.5)	2343 (9.6)
Blood pressure lowering (C02, C03, C08, C09)	1096 (34.3)	8293 (33.9)
Immunosuppressive (H02, L01, L04)	473 (14.8)	2548 (10.4)
Estrogen (G03A, G03C, L02A)	189 (5.9)	1469 (6.0)
Imaging		
Proportion undergoing imaging within 30 days	2355 (73.7)	4793 (19.6)
Positive imaging result (among those examined)	1730 (54.1)	0 (0) by definition

Values are mean ± SD, n (%), or median (IQR) as indicated

For laboratory variables, the number of patients in whom the value was available is shown in parenthesis after the result

B, blood, CRP, C-reactive protein, P, plasma, VTE, venous thromboembolism

**Table 2:** Factors predicting a diagnosis of venous thromboembolism within 1 year from the visit to the emergency department among 27,647 patients presenting with extremity symptoms

	HR (95% CI)	*P*-value	Adjusted HR (95% CI)	*P*-value
Age	1.011 (1.010–1.013)	< 0.001	0.994 (0.985–1.002)	0.135
Sex				
Male	1.00		1.00	
Female	0.899 (0.839–0.964)	0.003	0.996 (0.744–1.331)	0.976
Symptom category				
Unspecified	1.00		1.00	
Pain	0.626 (0.578–0.678)	< 0.001	0.820 (0.609–1.104)	0.190
Swelling	1.329 (1.213–1.457)	< 0.001	0.892 (0.639–1.244)	0.500
Laboratory parameters				
B-hemoglobin	1.005 (1.003–1.008)	< 0.001	1.005 (0.996–1.013)	0.287
P-creatinine	0.998 (0.998–0.999)	< 0.001	0.999 (0.994–1.004)	0.732
B-leucocyte count	0.983 (0.970–0.995)	0.007	0.978 (0.935–1.022)	0.318
B- platelet count	0.999 (0.999–1.000)	0.004	1.000 (0.998–1.002)	0.928
P-CRP				
< 5.0 mg/l	1.00		1.00	
≥ 5.0 mg/l	1.356 (1.249–1.473)	< 0.001	1.210 (0.894–1.639)	0.217
P-d-dimer				
< 0.5 mg/l	1.00		1.00	
≥ 0.5 mg/l	5.453 (4.552–6.533)	< 0.001	2.602 (1.894–3.575)	< 0.001
Recent (within 30 days) inpatient surgery				
No	1.00		1.00	
Yes	1.671 (1.487–1.877)	< 0.001	1.188 (0.784–1.798)	0.416
Comorbidities				
Malignancy				
No	1.00		1.00	
Yes	1.606 (1.472–1.752)	< 0.001	1.009 (0.711–1.431)	0.961
Ischemic heart disease				
No	1.00		1.00	
Yes	0.814 (0.717–0.926)	0.002	0.994 (0.569–1.498)	0.747
Other heart disease				
No	1.00		1.00	
Yes	0.719 (0.647–0.798)	< 0.001	0.957 (0.634–1.446)	0.835
VTE				
No	1.00		1.00	
Yes	16.606 (15.372–17.940)	< 0.001	6.037 (4.465–8.162)	< 0.001
Inflammatory disease				
No	1.00		1.00	
Yes	0.777 (0.683–0.884)	< 0.001	0.801 (0.481–1.331)	0.391
Diabetes mellitus				
No	1.00		1.00	
Yes	0.803 (0.708–0.910)	0.001	0.928 (0.548–1.570)	0.781

Only factors significant in univariate analysis were included in the adjusted analysis, which was performed in patients in whom all laboratory variables were available (*n =* 1122). Cox regression

B, blood, CI, confidence interval, CRP, C-reactive protein, HR, hazard ratio, P, plasma, VTE, venous thromboembolism

### Prediction of imaging for VTE

Among all included patients, 7,148 (25.9%) underwent diagnostic imaging for suspected VTE at or within 30 days of the first ED visit (Fig. 1). Baseline data in patients undergoing or not undergoing imaging are shown in Table 3. Table 4 shows that higher age (OR 1.007 [95% CI 1.000–1.013]; *p* = 0.002), extremity swelling (OR 1.445 [95% CI 1.009–1.941]; *p* = 0.011), higher d-dimer (OR 5.929 [95% CI 4.389–8.009]; *p* < 0.001), and previous VTE (OR 2.835 (95% CI 2.147–3.745]; *p* < 0.001) independently predicted a clinical decision of imaging in adjusted analysis.

**Table 3:** Baseline data in patients undergoing and not undergoing diagnostic imaging for suspected venous thromboembolism at and during 30 days after the visit to the emergency department among 27,647 patients presenting with extremity symptoms

Baseline variable	Imaging within 30 days (*n =* 7148)	No imaging within 30 days (*n =* 20,499)
Age (years)	64.0 ± 17.5	58.6 ± 19.8
Male sex	3310 (46.3)	9957 (48.6)
Symptom category pain	3160 (44.2)	12,113 (59.1)
Symptom category swelling	1911 (26.7)	2354 (11.5)
Symptom category unspecified	2077 (29.1)	6032 (29.4)
B-hemoglobin (g/l)	134 ± 18 (*n =* 5545)	134 ± 18 (*n =* 7821)
P-CRP (mg/l)	6.3 (1.8–27.0, *n =* 3129)	7.2 (2.2–29.0, *n =* 10,633)
B-leukocyte count (× 10^9^/l)	8.1 (6.6-.9.8, *n =* 4205)	8.8 (7.1–11.0, *n =* 6046)
B-platelet count (× 10^9^/l)	254 ± 88 (*n =* 4369)	257 ± 88 (*n =* 5010)
P-creatinine (micromol/l)	83 ± 44 (*n =* 5573)	90 ± 65 (*n =* 7831)
P-D-dimer (mg/l)	0.13 (0.1–0.33, *n =* 656)	0.16 (0.10–0.40, *n =* 2125)
Recent (30 days) outpatient surgery (n[%])	979 (13.7)	2746 (13.4)
Recent (30 days) inpatient surgery (n[%])	625 (8.7)	1068 (5.2)
Comorbidities (ICD codes)		
Malignancy (C)	1328 (18.6)	2345 (11.4)
Hypertensive disease (I10-16)	1918 (26.8)	4870 (23.8)
Ischemic heart disease (I20-25)	703 (9.8)	1958 (9.6)
Other heart disease (I30-50)	1099 (15.4)	3504 (17.1)
Cerebrovascular disease (I60-69)	412 (5.8)	1069 (5.2)
VTE (I26, 80–82)	2389 (33.4)	1321 (6.4)
Coagulation diseases (D65-69)	196 (2.7)	335 (1.6)
Inflammatory disease (M04-14, 30–36)	622 (8.7)	2091 (10.2)
Alcohol abuse (F10)	150 (2.1)	611 (3.0)
Diabetes mellitus (E08-13)	737 (10.3)	2094 (10.2)
Ongoing medication (ATC codes)		
Anticoagulation or antiplatelet (B01)	2431 (34.0)	5036 (24.6)
Lipid lowering (C10)	1258 (17.6)	3565 (17.4)
Glucose lowering (A10)	668 (9.3)	1916 (9.3)
Blood pressure lowering (C02, C03, C08, C09)	2660 (37.2)	6729 (32.8)
Immunosuppressive (H02, L01, L04)	913 (12.8)	2108 (10.3)
Estrogen (G03A, G03C, L02A)	459 (6.4)	1199 (5.8)

Values are mean ± SD, n (%), or median (IQR) as indicated

For laboratory variables, the number of patients in whom the value was available is shown in parenthesis after the result

B, blood, CRP, C-reactive protein, HR, hazard ratio, P, plasma, VTE, venous thromboembolism

**Table 4:** Factors predicting clinical need for imaging regarding venous thromboembolism within 30 days from the visit to the emergency department among 27,647 patients presenting with extremity symptoms

	OR (95% CI)	*P*-value	Adjusted OR (95% CI)	*P*-value
Age	1.015 (1.013–1.016)	< 0.001	1.007 (1.000–1.013)	0.002
Sex				
Male	1.00		1.00	
Female	1.095 (1.038–1.156)	< 0.001	0.990 (0.793–1.236)	0.930
Symptom category				
Unspecified	1.00		1.00	
Pain	0.758 (0.711–0.807)	< 0.001	1.108 (0.861–1.426)	0.425
Swelling	2.358 (2.180–2.550)	< 0.001	1.445 (1.009–1.941)	0.011
Laboratory parameters				
P-creatinine	0.997 (0.996–0.988)	< 0.001	1.001 (0.998–1.004)	0.537
B-leucocyte count	0.959 (0.948–0.970)	< 0.001	1.010 (0.984–1.037)	0.458
P-d-dimer				
< 0.5 mg/l	1.00			
≥ 0.5 mg/l	6.900 (5.599–8.504)	< 0.001	5.929 (4.389–8.009)	< 0.001
Recent (within 30 days) inpatient surgery				
No	1.00		1.00	
Yes	1.743 (1.573–1.932)	< 0.001	1.350 (0821–2.218)	0.327
Comorbidities				
Malignancy				
No	1.00		1.00	
Yes	1.766 (1.641–1.901)	< 0.001	1.154 (0.818–1.626)	0.415
Hypertensive disease				
No	1.00		1.00	
Yes	1.177 (1.107–1.251)	< 0.001	0.993 (0.744–1.324)	0.960
Other heart disease				
No	1.00		1.00	
Yes	0.881 (0.818–0.949)	0.001	0.716 (0.508–1.011)	0.058
VTE				
No	1.00		1.00	
Yes	7.268 (6.766–7.850)	< 0.001	2.835 (2.147–3.745)	< 0.001
Coagulation disorders				
No	1.00		1.00	
Yes	1.697 (1.420–2.028)	< 0.001	2.332 (0.976–5.576)	0.057
Inflammatory disease				
No	1.00		1.00	
Yes	0.839 (0.764–0.922)	< 0.001	1.048 (0.708–1.549)	0816
Alcohol abuse				
No	1.00		1.00	
Yes	0.698 (0.582–0.836)	< 0.001	0.771 (0.363–1.636)	0.497

Only factors significant in univariate analysis were included in the adjusted analysis, which was performed in patients in whom all laboratory variables were available (*n =* 1728)

B, blood, CI, confidence interval, CRP, C-reactive protein, OR, odds ratio, P, plasma, VTE, venous thromboembolism

### Prediction of imaging results

Among the 7148 patients undergoing diagnostic imaging at or within 30 days of the ED visit, 1730 (24.2%) were diagnosed with VTE (Fig. 1). Table 5 shows baseline data in those with or without VTE on imaging. As evident in supplementary Table 1, lower age (OR 0.984 [95% CI 0.972–0.997]; *p* = 0.014), higher hemoglobin (OR 1.023 [95% CI 1.010–1.037]; *p* < 0.001), higher CRP (OR 2.229 [95% CI 1.433–3.468]; *p* < 0.001), and higher d-dimer (OR 8.729 [95% CI 5.614–13.574]; *p* < 0.001) levels, as well as a previous VTE diagnosis (OR 7.796 [95% CI 5.193–11.705]; *p* < 0.001) independently predicted a positive finding on imaging in adjusted analysis, whereas female sex (OR 0.602 [95% CI 0.392–0.924]; *p* = 0.020) and a previous diagnosis of ischemic heart disease (OR 0.254 [95% CI 0.113–0.571]; *p* = 0.001), were negative predictors of VTE.

**Table 5:** Baseline data among 7148 patients undergoing imaging for venous thromboembolism at and up to 30 days after the visit at an emergency department with extremity symptoms

Baseline variable	VTE on imaging within 30 days (*n =* 1730)	No VTE on imaging within 30 days (*n =* 5418)
Age (years)	65.1 ± 16.7	63.6 ± 17.7
Male sex	954 (55.1)	2356 (43.5)
Symptom category pain	700 (40.5)	2460 (45.4)
Symptom category swelling	518 (29.9)	1393 (25.7)
Symptom category unspecified	512 (29.6)	1565 (28.9)
B-hemoglobin (g/l)	136 ± 19 (*n =* 1569)	134 ± 17 (*n =* 3976)
P-CRP (mg/l)	7.6 (2.0–32.0, *n =* 1573)	6.1 (1.8–25.0, *n =* 4000)
B-leukocyte count (× 10^9^/l)	9.1 ± 9.5 (*n =* 1142)	8.7 ± 6.0 (*n =* 3063)
B-platelet count (× 10^9^/l)	246 ± 94 (*n =* 1422)	258 ± 85 (*n =* 2947)
P-creatinine (micromol/l)	84 ± 37 (*n =* 1573)	82 ± 46 (*n =* 4000)
P-D-dimer (mg/l)	0.12 (0.10–0.35, *n =* 159)	0.14 (0.10–0.33, *n =* 527)
Recent (30 days) outpatient surgery (n[%])	257 (14.9)	722 (13.3)
Recent (30 days) inpatient surgery (n[%])	176 (10.2)	449 (8.3)
Comorbidities (ICD codes)		
Malignancy (C)	386 (22.3)	942 (17.4)
Hypertensive disease (I10-16)	420 (24.3)	1498 (27.6)
Ischemic heart disease (I20-25)	112 (6.5)	591 (10.9)
Other heart disease (I30-50)	211 (12.2)	888 (16.4)
Cerebrovascular disease (I60-69)	94 (6.4)	318 (5.9)
VTE (I26, 80–82)	1246 (72.0)	1143 (21.1)
Coagulation diseases (D65-69)	55 (3.2)	141 (2.6)
Inflammatory disease (M04-14, 30–36)	125 (7.2)	497 (9.2)
Alcohol abuse (F10)	36 (2.1)	114 (2.1)
Diabetes mellitus (E08-13)	137 (7.9)	600 (11.1)
Ongoing medication (ATC codes)		
Anticoagulation or antiplatelet (B01)	771 (44.6)	1600 (30.6)
Lipid lowering (C10)	237 (13.7)	1021 (18.8)
Glucose lowering (A10)	130 (7.5)	538 (9.9)
Blood pressure lowering (C02, C03, C08, C09)	588 (34.0)	2072 (38.2)
Immunosuppressive (H02, L01, L04)	262 (15.1)	651 (12.0)
Estrogen (G03A, G03C, L02A)	105 (6.1)	354 (6.5)

Values are mean ± SD, n (%), or median (IQR) as indicated

For laboratory variables, the number of patients in whom the value was available is shown in parenthesis after the result

B, blood, CRP, C-reactive protein, HR, hazard ratio, P, plasma, VTE, venous thromboembolism

### Later VTE among patients negative at imaging within 30 days

Among patients negative at imaging within 30 days after the initial visit, those with a later VTE diagnosis between 30 days and one year more often had higher d-dimer at baseline, previous VTE, and ongoing anticoagulation compared to those remaining free from VTE (Supplementary Table 2).

## Discussion

Among all studied patients, 11.6% were diagnosed with VTE within one year of the initial ED visit, and only an increased d-dimer and a previous diagnosis of VTE independently predicted a VTE diagnosis within one year. Three out of four patients diagnosed with VTE within one year had undergone imaging already at or within 30 days after the ED visit, and half of the patients had received their VTE diagnosis at this examination. A substantial number of VTE diagnoses were thus made after one month, both in patients with negative imaging within 30 days and patients not undergoing imaging within 30 days. These figures of course do not necessarily reflect diagnostic negligence at the ED; a substantial number of the diagnoses made more than 30 days after the ED visit might reflect new episodes of VTE. These findings highlight the need for continued vigilance on extremity and pulmonary symptoms and wide indications for appropriate repeated diagnostic imaging in such patients. As well-established risk factors [12] such as an increased d-dimer and a previous diagnosis of VTE independently predicted a VTE diagnosis within one year, the clinician should pay special attention to patients with these features. This is corroborated by the subgroup analysis of patients with negative imaging within 30 days, showing that patients later developing VTE had higher d-dimer at baseline, more prior VTE episodes, and a higher proportion of ongoing anticoagulation treatment.

Furthermore, the study showed that every fourth patient underwent imaging for suspected VTE at or during the first 30 days after their first ED visit for swelling, pain, or other unspecified symptoms from the extremities. Several factors at presentation, such as higher age, extremity swelling, higher d-dimer, and previous VTE, independently predicted diagnostic imaging for suspected VTE, either DVT or PE, within 30 days from the ED visit. This can be expected since the most validated risk score for DVT recommended [19] in Region Skåne, the Wells score [9–11], includes both extremity swelling and previous VTE in the estimation of the likelihood of DVT. Furthermore, the Wells score recommends a d-dimer test in subjects with a low VTE likelihood, and imaging when this test is positive [9–11]. Among the other components in the Wells score [9–11] however, recent surgery or previous malignancy were not predictive of imaging.

Among the subgroup of patients undergoing imaging, a VTE diagnosis was made within 30 days in every fourth patient, corresponding to 6.3% of the total number of patients with extremity symptoms. This proportion is lower than the previously reported VTE rate of 10–15% within three months among patients with suspected VTE, either DVT or PE [8, 20]. In the 6 studies on DVT with a total of 3,875 patients included in the meta-analysis by Ten Cate-Hoek [20] however, the suspicion of VTE was more distinctly formulated than in our sample of patients with a broader range of extremity symptoms. Among the quartile of patients undergoing imaging within 30 days from the ED visit, lower age, higher hemoglobin, CRP, and d-dimer, and previous VTE predicted a VTE finding at investigation, whereas female sex and ischemic heart disease were protective. Again, recent surgery or previous malignancy was not predictive of the imaging results. This corroborates that symptoms, signs, and risk factors cannot be individually used to identify a DVT, and further strengthens the rationale for using the Wells score [9–11], which takes many risk factors into account. In this context, however, variables not included in the Wells score [9–11] such as higher hemoglobin and CRP-levels also predicted a positive imaging result, suggesting a relevance of viscosity and inflammation for thrombosis formation. No such associations were demonstrated in a study on the VTE risk in healthy middle-aged men [21], but this patient group is different from the presently studied ED cohort.

The concept of AI and machine learning-based diagnostic algorithms for decision-making [13] has been evaluated in VTE [13], and a recent meta-analysis [22] identified 12 previous studies in this field. The models used and types of VTE explored varied widely. AI models were for example evaluated to predict cancer associated thrombosis [23], and to predict outcome in critically ill patients with VTE [24]. Only one study evaluated prediction of VTE in the ED setting [25]. In this study of 3145 ED patients with suspected VTE, patients with low pretest probability were followed for 90 days [25]. The study largely focused on prediction of PE and in addition to factors assessed in our analysis, such as age, sex, previous VTE, malignancy, and estrogen use, the model included several other variables relevant for PE prediction, such as pulse rate, blood pressure, respiratory rate, oxygen saturation, and body temperature. Such information was not systematically collected in our population presenting with pain, swelling or other symptoms from the extremities, however.

The field of prediction and diagnosis of DVT and VTE is wide and complex, and factors predictive of VTE presumably vary widely between outpatients at an ED and inpatients with concomitant predisposing diseases [24]. AI models are promising but must be tailored for the different diagnostic settings before widespread use in clinical practice. Whether other variables could be predictive in non-linear AI-models for VTE prediction has to be further studied. Genomic markers might also prove to be of diagnostic value [26], but their utility is currently limited as results are not available rapidly enough to enable decisions in the ED setting. In the meantime, we should rely on the well-established risk factors already included in diagnostic algorithms for VTE prediction [6–11]. But despite both international [12] and local [19] recommendations, the current study showed that the Wells score was not always used in routine clinical practice.

The present study has several other important limitations, such as its retrospective and non-randomized design. Adjusted OR could also only be calculated upon patients in whom all relevant laboratory data were available. Furthermore, the RETTS system [14, 15] classifies patients as having either pain, swelling, or unspecified symptoms from the extremities, but does not separate patients with leg or arm symptoms. The predictive variables for both imaging and diagnosis of VTE might of course differ substantially in these situations. It would of course also have been preferable if all components of the Wells score [9–11] had been systematically assessed and electronically documented for evaluation in the subgroup with symptoms from the legs. In addition to the missing components in the Wells score, such as entire leg swelling, tenderness, pitting edema, and collaterals, information is lacking regarding body temperature, blood pressures, and pulse rates which might have been relevant for the risk of PE. Only patients presenting with extremity symptoms were included, and hence DVT at more rare locations such as intraabdominally or in the central nervous system was not evaluated. Furthermore, only patients residing in Sweden on December 31, the year before their ED visit, were included, and subjects with a VTE moving abroad after the visit might thus have been missed.

DVT and PE are different manifestations of the same disease entity, and 30–66% of DVT patients have findings on CT or scintigraphy suggesting clinically silent PE [27–29]. The fact that patients were evaluated not only regarding imaging and imaging results for DVT, but also for PE therefore represents a strength of the study. Other important strengths are the size of the material, and the coverage of all EDs in the region, in which diagnoses of VTE in outpatients are most often made and always confirmed at a hospital ED [20] and not in primary care. The use of national Swedish registry data for follow-up has been validated and is considered reliable [16].

In conclusion, a large amount of information is collected for each patient presenting to the ED with extremity symptoms suggesting DVT. This analysis in > 27,000 patients with extremity symptoms confirmed that well-established risk factors for VTE already included in diagnostic algorithms could be used to predict VTE in an ED setting. A large proportion of study patients with extremity symptoms who developed VTE within one year had negative diagnostic imaging within 30 days of the ED visit, illustrating the importance of continued vigilance for symptoms within this group.

## Conflict of interest

AG is supported by Research funds at Skåne University Hospital, Region Skåne, and the Hulda Ahlmroth Foundation. He has received consulting fees from Sanofi, Bayer and Pfizer. UE, OM, AB, and BO report no conflict of interest.

## Data Availability

All datafiles supporting the results reported in the article are available from the corresponding author upon reasonable request.
